# Supplementary material for: Adjuvant Icotinib in EGFR-mutated stage IB non-small cell lung cancer with high-risk factors: A retrospective case series
Source: PLoS One. 2025 Nov 19;20(11):e0337237. doi: 10.1371/journal.pone.0337237 (PMC12629473; doi:10.1371/journal.pone.0337237)
Supplement: Attachment — Submitted filename: Response to Reviewers.docx [file pone.0337237.s002.docx]

****Dear Editors and Reviewers,****

**We sincerely thank you and all reviewers for your valuable time and insightful feedback on our manuscript. We appreciate the opportunity to revise our work based on your constructive comments, which have significantly enhanced the manuscript and provided valuable inspiration for our future research endeavors. Please convey our gratitude to the reviewers. In response to the reviewers' comments, we have carefully revised the manuscript. We hope the revised version now meets the journal's standards. Below, we provide point-by-point responses to each comment and question.**

****To Reviewer #1****

**We are grateful for your critical evaluation and thoughtful suggestions, which have been instrumental in improving our manuscript. We have thoroughly addressed each point raised. Below are our detailed responses.**

**Comment 1:** The first part of the result section can be visualized using demographic related figs.
****Response:** Thank you for highlighting this point. We have incorporated figures to visualize the demographic data presented in the first part of the results section, and the manuscript has been updated accordingly.**

**Comment 2:** The authors mentioned that chest computed tomography (CT), cranial magnetic resonance imaging (MRI), hepatic and adrenal ultrasounds, and tumor markers detection were conducted every three months to evaluate the outcomes. Providing representative follow-up MRI or CT images at different time points (before, during and after treatment) for patients responsive and resistant to alectinib can complement current provided results.
****Response:** **W**e appreciate this suggestion. As this study exclusively involved patients receiving adjuvant alectinib therapy following complete resection (stage IB), no post--treatment radiological assessments for response evaluation (as would be typical in neoadjuvant or metastatic settings) were performed. The imaging conducted every three months was for post-treatment surveillance of recurrence. Consequently, images depicting "before" or "during" treatment response are not available. However, as suggested, representative radiological images (CT/MRI) demonstrating recurrence in two patients have now been included as supplementary material.**

**Comment 3:** Confidence interval and p-value, log-rank or Cox regression not mentioned for DFS data.
****Response:** We thank you for raising this methodological point. Given that this is a single-arm cohort study without a comparator group, formal statistical comparisons (e.g., log-rank test, Cox regression) for DFS were not applicable, and thus p-values and hazard ratios could not be calculated. The analysis presented is descriptive, reporting the observed DFS rates with confidence intervals and events.**

**Comment 4:** What method was used for EGFR mutation detection? blood or tissue?
****Response:** EGFR mutation status was confirmed using a 10-gene next-generation sequencing (NGS) panel based on PCR-ARMS technology. This testing was performed on **tumor tissue** samples obtained at the time of surgical resection.**

**Comment 5:** The readability of paragraphs can be improved.
****Response:** We thank you for this essential feedback. The language and readability throughout the manuscript have been carefully revised.**

**Comment 6:** Was radiologic response assessed (e.g., RECIST criteria)? if yes please explain.
****Response:** We appreciate this query. Consistent with the study design focusing on adjuvant therapy after complete resection (stage IB disease), patients did not have measurable disease at the start of icotinib treatment. Therefore, formal radiologic response assessment using RECIST criteria was **not applicable** in this setting.**

****To Reviewer #2****

**We sincerely appreciate your critical review and valuable suggestions. We have carefully considered each comment and revised the manuscript accordingly. Our detailed responses are provided below:**

**Comment 1:** Please indicate the time interval between surgical resection and initiation of adjuvant therapy.
****Response:** Thank you for this request for clarification. The adjuvant alectinib therapy was initiated **one month** after surgical resection.**

**Comment 2:** With the exception of patients who experienced a recurrence, did all others complete the 36-month treatment period? If not, please indicate whether any sub–grade 3 adverse events led to treatment discontinuation or modification, and specify the patient(s) involved.
****Response:** We thank you for this important question. Not all patients who did not experience recurrence completed the full 36-month treatment period. The median duration of treatment was 30 months (range 18-36 months) at the time of data cutoff. However, **no** discontinuation of treatment or dose reduction was necessitated by adverse events of grade<3. Treatment modifications or discontinuations, where they occurred, were unrelated to toxicity below grade 3 severity.**

**Comment 3:** Kindly revise the number of sublobectomies shown in Table 1, if inaccurate.
****Response:** Thank you for highlighting this point. The number of sublobectomies presented in Table 1 was indeed inaccurate and has been corrected accordingly in the revised manuscript and table 1.**
