# Supplementary material for: Adjuvant Icotinib in EGFR-mutated stage IB non-small cell lung cancer with high-risk factors: A retrospective case series
Source: PLoS One. 2025 Nov 19;20(11):e0337237. doi: 10.1371/journal.pone.0337237 (PMC12629473; doi:10.1371/journal.pone.0337237)
Supplement: Attachment — Submitted filename: Response_to_Reviewers_auresp_2.docx [file pone.0337237.s003.docx]

**Dear Editors and Reviewers,**

**We sincerely thank you and all reviewers for your valuable time and insightful feedback on our manuscript. We appreciate the opportunity to revise our work based on your constructive comments, which have significantly enhanced the manuscript and provided valuable inspiration for our future research endeavors.**

**Please convey our gratitude to the reviewers. In response to the reviewers' comments, we have carefully revised the manuscript. We hope the revised version now meets the journal's standards. Below, we provide point-by-point responses to each comment and question.**

**To Reviewer #3,**

**We are grateful for your critical evaluation and thoughtful suggestions, which have been instrumental in improving our manuscript. We have thoroughly addressed each point raised. Below are our detailed responses.**

**Comment 1:** There are still some grammatical errors but the manuscript is able to be read.
**Response:** **We thank you for this essential feedback. We have undertaken a comprehensive language polish of the manuscript, which involved not only correcting grammatical errors but also refining word choice, sentence structure, and overall clarity of expression to enhance readability. All modifications have been highlighted in red in the revised manuscript file. We hope the language quality of the manuscript is now significantly improved.**

**Comment 2:** Line 58 Intro: Update to 2024 or estimated 2025 figures.
**Response:** **We appreciate this suggestion. We have thoroughly searched for the GLOBOCAN 2024 or 2025 estimates. However, as of the preparation of this revision (September 2025), the most recent comprehensive global cancer incidence and mortality data available from the WHO/IARC is still the GLOBOCAN 2022 set, published in 2024. Therefore, we have maintained this citation and slightly rephrased the sentence to clarify the timeliness of the data source.**

**Comment 3:** Rather than focusing on just enlarging the sample, adding a cohort of matched controls with similar features could assist.
**Response:** **We thank the reviewer for this exceptionally constructive suggestion. We completely agree that the absence of a matched control cohort is a key limitation of our current retrospective study. In direct response to this comment, we have now explicitly acknowledged this limitation in the 'Discussion' section and have initiated plans to conduct a follow-up case-control study to address it in future work.**

**Comment 4:** The vast majority of patients had VPI as their adverse feature. It is not clear if the high disease control rate would extend to the other high risk features his should be addressed in the discussion as well of any relevant adverse features that were published in the sited series.
**Response:** **We thank the reviewer for raising this critical point.** **The reviewer is absolutely correct that the vast majority (91.9%) of our cohort presented with visceral pleural invasion (VPI). Consequently, our study is underpowered to perform a statistically meaningful subgroup analysis to determine whether the high disease-control rate extends equally to patients with other high-risk features, given the very low number of events within these small subgroups. Furthermore, upon reviewing the literature, we found limited evidence evaluating the prognostic influence of these specific high-risk features in the context of postoperative adjuvant targeted therapy. This gap further highlights the significance of our findings. Meanwhile, to address this limitation, we have cited relevant publications (e.g., [13-15, 18-19]) in the Discussion, affirming that features such as lymphovascular invasion, spread through air spaces, and poor differentiation are well-established independent adverse prognostic factors in early-stage NSCLC.**

**Comment 5:** Would include stage II to IIIA, EGFR-mutated, non–small cell lung cancer adjuvant icotinib III trial (ICTAN, GASTO1002) presented at the 2024 American Society of Clinical Oncology (ASCO) Annual Meeting (Abstract 8004) to highlight that the optimal duration of use is unknown (even though these patients were stage II and III. This is relevant as the CORIN trial sited also has a more limited duration of therapy- 12 months. The ADAURA study, however, selected a 36 month duration of therapy which was the recommended duration of therapy your patients were receiving.
**Response:** **We sincerely thank the reviewer for this critical and highly insightful comment, which has significantly improved the depth of our discussion. As suggested,** **we have included a discussion of the ICTAN trial findings in the Introduction section of our revised manuscript. Furthermore, we fully agree that the optimal duration of adjuvant TKI therapy remains a pivotal clinical question. To address this, we have expanded the Discussion section to explicitly acknowledge this uncertainty, citing the differing durations used in the CORIN (12 months), ICTAN (6-12 months), and ADAURA (36 months) trials. We clarify that the 36-month regimen used in our study was aligned with the ADAURA protocol and current clinical guidelines at the time of treatment. We also emphasize that future studies are needed to definitively establish the optimal treatment duration.**
